# Exploiting Bacterial Pigmentation for Non-Destructive Detection of Seed-Borne Pathogens by Using Photoacoustic Techniques

**DOI:** 10.3390/s24237616

**Published:** 2024-11-28

**Authors:** Lucia Cavigli, Dario Gaudioso, Cecilia Faraloni, Giovanni Agati, Stefania Tegli

**Affiliations:** 1 Consiglio Nazionale delle Ricerche, Istituto di Fisica Applicata “Nello Carrara”, Via Madonna del Piano 10, 50019 Sesto Fiorentino, Italy; l.cavigli@ifac.cnr.it; 2 Dipartimento di Scienze e Tecnologie Agrarie, Alimentari Ambientali e Forestali, Laboratorio di Patologia Vegetale Molecolare, Università degli Studi di Firenze, Via della Lastruccia 10, 50019 Sesto Fiorentino, Italy; dario.gaudioso@unifi.it; 3 Consiglio Nazionale delle Ricerche, Istituto per la BioEconomia, Via Madonna del Piano 10, 50019 Sesto Fiorentino, Italy; cecilia.faraloni@ibe.cnr.it

**Keywords:** absorbance spectra, bacterial pigments, *Curtobacterium flaccumfaciens* pv. *flaccumfaciens*, leguminosae, molecular diagnostics, photoacoustics, quarantine pathogens, seed-borne pathogens

## Abstract

Seed-borne pathogens pose a significant threat to global food security. This study focuses on *Curtobacterium flaccumfaciens* pv. *flaccumfaciens* (*Cff*), a quarantine plant pathogen causing bacterial wilt of common beans. Despite its global spread and economic impact, effective control measures are limited. Existing diagnostic methods, such as PCR, are time-consuming, destructive, and challenging for large-scale screening. This study explores the potential of photoacoustic techniques as a non-destructive, rapid, and high-throughput alternative. These techniques leverage the photoacoustic effect to measure optical absorption, offering high sensitivity and accuracy. *Cff* colonies exhibit distinct pigmentation, suggesting their suitability for photoacoustic detection. We characterised the optical properties of *Cff* and developed an in vitro model to simulate conditions within *Cff*-infected bean seeds. The results demonstrate the efficiency of the photoacoustic technique in detecting *Cff* in a mimicked-bean seed and indicate the potential discrimination of different coloured *Cff* strains. This study paves the way for a novel, non-invasive approach to the early detection of *Cff* and other seed-borne pathogens, contributing to improve crop health and food security.

## 1. Introduction

In a context where global food demand is continuously rising, the need for innovative, highly specific, sensitive, high-throughput, and low-cost assays for the early detection of seed-borne phytopathogens constitutes a key step to grow healthy and safe crops, save yield and profit, and reduce the use of plant protection products. Seed-borne pathogens may easily spread undetected, even at long distances and in countries where they were previously absent, by infected seeds that are very often asymptomatic [1,2,3]. Therefore, visual examination of seed lots is mostly useless, while cutting-edge diagnostics for stealth infections of seeds are urgently needed [4].

Here, we focused on a seed-borne Gram-positive bacterium that is among the quarantine plant pathogens for the European Union (EU) [5,6] called *Curtobacterium flaccumfaciens* pv. *flaccumfaciens* (Hedges) Collins & Jones (hereafter, *Cff*). This aerobic, non-spore-forming bacterium is the causal agent of the so-called “bacterial wilt of bean” and “tan spot disease of soybean”. *Cff* is one of the most important bacterial constraints affecting the production of edible legumes worldwide [7,8], which, together with cereals, are the other most important staple food [9,10].

Despite the categorisation of *Cff* as a quarantine plant pathogen in many countries and continents, this bacterium still continues to spread worldwide and cause heavy yield and economic losses in the most important bean-producing countries, such as Brazil, Canada, eastern Australia, Iran, and USA [6,8].

As no effective chemicals against this bacterium are known and available yet, the development of highly specific and sensitive diagnostic tests for the rapid detection of *Cff* on seed lots and other plant materials is pivotal to prevent *Cff* disease outbreaks. Currently, the visual inspection of suspected seed lots can be complemented by some officially recognised molecular PCR-based methods highly specific for *Cff* detection [11,12,13], to be performed even on-site, such as a “loop-mediated isothermal amplification” (LAMP) assay [14]. These PCR-based tests are highly targeted on any *Cff* variant discovered so far, but they have several important intrinsic limits, such as being destructive procedures, quite time-consuming, and laborious, heavily challenging for a statistically reliable sampling.

Accordingly, non-destructive and rapid diagnostic tests are of great interest, such as those based on the detection of specific volatile organic compounds (VOCs) [15,16,17], or on several spectroscopic approaches [4,18,19].

The most relevant examples of optical technologies employed for plant disease diagnosis were mainly based on Vis-NIR reflectance spectroscopy [18,20,21]. Their main shortcoming consists of the limited penetrating power of the probe beans [18,22]. Raman spectroscopy exploits laser (Vis or NIR) irradiation and no sample preparation is needed [23]. However, just in a few cases, it was successfully applied to detect pathogens in seeds and grains [20,24,25]. The main drawback of this technique is represented by the presence in biological samples of a significant background due to fluorescence that can negatively affect the Raman spectra.

Photoacoustic spectroscopy (PAS) [26,27] has also recently emerged as a novel technology in plant and agricultural sciences to study various physiological processes and stress responses [28,29,30], even if its application in seed health testing remains limited [31,32]. PAS leverages the photoacoustic (PA) effect to measure the optical absorption by a sample, which then generates acoustic waves. This method is particularly sensitive and can determine absorption coefficients more accurately than conventional spectroscopy [26]. However, one of the main challenges with PAS is that many setups rely on indirect gas-phase cell detection, which is not practical for field use [26].

Recent advancements have focused on the direct detection of laser-induced ultrasound waves, particularly in biomedical imaging [33,34,35]. In PA imaging, ultrasound waves are excited by irradiating specific chromophores with a pulsed laser (usually on a nanosecond timescale). The light absorption followed by rapid conversion to heat produces a small temperature rise which leads to an initial pressure increase, which subsequently results in the emission of broadband ultrasound waves. The latter propagate in the medium and can be directly detected by an ultrasound receiver to reconstruct images based on the optical absorption properties of the sample [33].

While these direct detection methods have shown great promise in the biomedical field [33,34,35], their potential in agri-food applications is still being unexplored [36]. This could open up new avenues for non-invasive diagnostics in plant health and crop monitoring.

*Cff* was also defined as “the multicoloured bacterium” [37] because of the different strain-specific pigmentation that *Cff* colonies exhibit when grown in vitro, and of the staining that is sometimes shown by white-coated *Cff*-infected bean seeds. The presence of absorbers in the longer-wavelength range of the visible spectrum suggests evaluating the feasibility of a PA approach based on the direct detection of laser-induced ultrasound waves for the development of a non-destructive, high-throughput detection of asymptomatic *Cff* infections in bean seeds. Accordingly, here, we characterised the optical properties of *Cff* colonies, determined the main compounds contributing to absorbance, and applied the PA method on an in vitro model, based on innovative cultural media for *Cff* growth mimicking the conditions found by this seed-borne bacterium in nature.

## 2. Materials and Methods

### 2.1. Bacterial Strains and Growth Conditions

**Figure 1:**
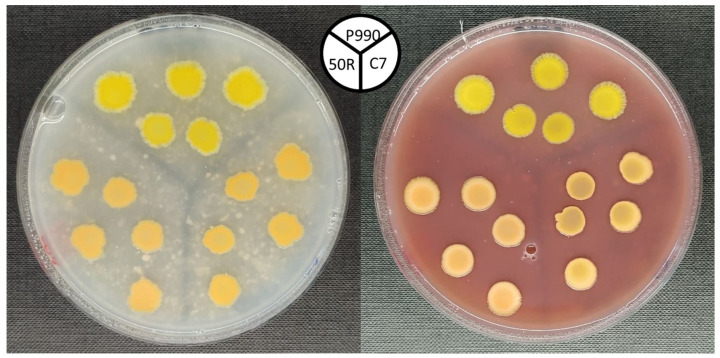
*Cff* strains grown on naturalised media, based on Cannellino (**left**) and on Borlotto bean flour (**right**).

The *Cff* bacterial strains used here, and their main features, are reported in Table 1. They include three representative and different colour variants. These are *Cff* P990, *Cff* 50R, and *Cff* C7, which are yellow-, red-, and orange-pigmented, respectively. For long-term storage, *Cff* strains were preserved in 40% w/v glycerol at −80 °C, and subcultured when needed. *Cff* strains were routinely grown on nutrient broth yeast extract medium [38] and on Luria Bertani (LB) medium [39] both as liquid and solid cultures and incubated at 26 °C. Moreover, in order to mimic the bean seed physico-chemical environment, the so-called “naturalised media” were newly developed here for *Cff* growth and PA analysis. Basically, Cannellino and Borlotto bean varieties were separately used to produce flour to be then used to support *Cff* growth. After several preliminary attempts, the final flour concentration in each naturalised medium was established to be 36 g/L. The different agarised media were then spotted with several drops (5 μL/each) of a fresh *Cff* bacterial suspension (OD_600_ = 0.8) (Figure 1). After an incubation at 26 °C for 14 days, *Cff* colonies were used as such for pigment extraction, while they were overlayed with the same medium previously used for their growth (20 mL/plate) to perform both optical and PA measurements (see Figure S1).

### 2.2. Chemical Extraction

After several preliminary experiments, the pigments produced by the *Cff* strains were extracted according to Grammbitter et al. (2019) [40], with some modifications. Bacteria were grown on agarised Cannellino naturalised medium, LB, and LB Sac10 (i.e., LB amended with 10% sucrose), incubated at 26 °C for 14 days. After that time, the bacterial biomass was harvested by picking up *Cff* colonies, washed once in sterile physiological solution (SPS) (0.85% NaCl w/v), and finally re-suspended in dimethyl sulfoxide (DMSO) (i.e., 50 mg of biomass for 200 μL of DMSO). Samples were then incubated in the dark at 50 °C on a rotary shaker (100 rpm) for 1.5 h. Subsequently, 200 μL of methanol was added to each sample and then thoroughly vortexed. The extracts were then centrifuged at 13,000 rpm for 15 min. The resulting supernatants were then centrifuged again at 13,000 rpm for 15 min, before measuring their absorbance spectra (Figure S2) by using 0.2 × 1 cm quartz cuvettes.

### 2.3. Pigment Determination

The high-performance liquid chromatography (HPLC) analysis of *Cff* pigments was carried out using a multi-solvent pump (ProStar 210, Varian Medical Systems, Inc., Las Vegas, NV, USA) and a photodiode array detector (Varian ProStar 335) [41]. The stationary phase consisted of a Phenomenex Kinetex Phenyl-Hexyl 100 A 150 × 4.6 mm reverse-phase C18 column with an identical pre-column operating at 25 °C.

The reagents used here for analysis were HPLC-grade, except tetrabutyl ammonium acetate salt solution, pH 6.5, which was prepared with distilled water and filtered. Eluent solutions were as follows: solvent A, 80:20 (v/v) methanol, 28 mM tetrabutyl ammonium acetate; solvent B, 100% methanol. For the elution, with a flow rate of 0.8 mL/min, a linear gradient was used as follows: starting with 100% of solvent A, 100% of solvent B was reached in 25 min, which was maintained for 2 min, and then 100% of solvent A was reached in 3 min. Pigment identification was based on the retention times and comparison of the absorption spectra to those of HPLC-grade carotenoid standards (Sigma-Aldrich, St. Louis, Missouri, USA), as well as on previously reported analyses [42,43].

### 2.4. Photoacoustic Setup

Photoacoustic tests were carried out on a home-made test bench mounted in an up-right transmittance configuration (Figure S3a) [44]. As the laser source, we used a supercontinuum pulsed laser (SuperK COMPACT, NKT Photonics A/S, Birkerød, Denmark) for broad-band irradiation (wavelength range 440–2400 nm, pulse duration 2 ns, repetition rate 10 kHz), focused on the samples by an objective lens (magnification 4×). Several glass filters were employed to select different band emissions: a coloured glass heat-absorbing short-pass filter (SCHOTT KG3, 50.8 × 50.8 mm, 3 mm thick) to cut the near-infrared laser component, and the kit of SCHOTT Coloured Glass Longpass Filters (50.8 × 50.8 mm, 3 mm thick, cut-on wavelength from 475 nm to 630 nm). The laser emission band and the filter transmittance spectra are shown in Figure S4. An amplified immersion transducer from Olympus Panametrics (mod V382-SU-F, frequency range 3.5 kHz, focal distance 0.83 inch, 40 dB amplifier mod 5676) was used as the ultrasound (US) receiver. For each laser pulse, the corresponding signal from the the US receiver was acquired with a Rohde & Schwarz oscilloscope, model RTO1004. A watertight tank, with an optical window on the bottom, allowed both samples and the transducer to be kept immersed in water.

The typical PA signal as detected with the oscilloscope is reported in Figure S3b. The y-axis represents the received pulse signal in mV, while the x-axis displays the travel time of the ultrasonic pulse triggered by the light excitation (time zero). The arrival time to the transducer of the US pulse determines the position of the optical absorber (considering the speed of sound in water of 1482 m $s^{-1}$ [45]). The PA signal’s amplitude is directly proportional to the incident light intensity and the target molecule absorbance, while the period of each PA pulse is influenced by the sample’s geometry, i.e., shorter periods correspond to smaller PA sources [33,46].

### 2.5. Optical Measurements

Total reflectance and transmittance measurements were performed with a spectrophotometer equipped with an integrating sphere (Jasco mod. 560-V, Jasco Corp., Tokyo, Japan). The same instrument was used to measure the absorbance spectra of methanolic bacteria extracts (Figure S2). For each *Cff* strain, the effective absorbance ($A_{\mathrm{eff}}$) of the bacteria colonies under different irradiation wavebands was calculated as the product between the *Cff* strain in vitro absorption spectrum (*A*) and the radiation spectrum impinging on the bacterial spot:

(1) $$A_{\mathrm{eff}}\left(\lambda \right)=A\left(\lambda \right)\;\cdot \;E_{L}\;\cdot \;T_{\mathrm{LP}}\;\cdot \;T_{\mathrm{SP}}\;\cdot \;T_{M},$$

where $E_{L}$ is the laser emission band; $T_{\mathrm{LP}}$ and $T_{\mathrm{SP}}$ are the transmittances of long-pass and short-pass filters, respectively; and $T_{M}$ the transmittance of the medium.

## 3. Results

### 3.1. *Cff* Colony Colour and Pigments Analysis

The aim of the development of innovative “naturalised” media here, based on bean flour, was to study and characterise the optical behaviour of *Cff* in an environment as close as possible to that of seeds. Preliminary experiments (data not shown) were carried out by using flour obtained from several legumes known to be a host for *Cff*. Then, common bean was selected because of many reasons: it is the major host for *Cff*, common bean seeds are the commodities subjected to phytosanitary inspections according to the current EU legislation on *Cff* as a quarantine pathogen [5,47], and seeds are strongly differently pigmented depending on the bean variety. To this concern, the Cannellino and Borlotto varieties have been selected because of their white- and red-pigmented seeds, respectively, determining very different optical features, which could be exploited during the in vitro model development for *Cff* detection in seeds. In particular, Cannellino and Borlotto are representative of a weak-absorbing and of a strong-absorbing host, respectively. For the first time, it was demonstrated here that *Cff* colony pigmentation is not just strain-specific, but it is also affected by the growth medium (Table 2). *Cff* pigmentation is strongly evident when bacteria are grown on naturalised media, while their colony colour decreases when grown on LB (Table 2). However, when sucrose was added (i.e., LBsac10), a stronger pigmentation was observed just by eye, with *Cff* P990 and C7 colonies shifting from straw-yellow and pale orange, respectively, to deep hue, while *Cff* 50R turned reddish (Table 2). Concerning pigmentation on bean-flour-based media, *Cff* P990 exhibited a brighter yellow colour when grown on naturalised media, *Cff* C7 showed different shades of orange depending on the bean flour used, and *Cff* 50R appeared less pigmented when incubated on Cannellino than on Borlotto flour, where the reddish colour was still evident (Table 2).

For the aim of our study, the full characterisation of pigments was not required, so we were limited to the determination of the absorption properties of the main compounds and their relative contribution in the samples investigated by PA. Therefore, colony pigments were separately extracted from *Cff* P990, *Cff* 50R, and *Cff* C7 strains and analysed, as described above.

**Figure 2:**
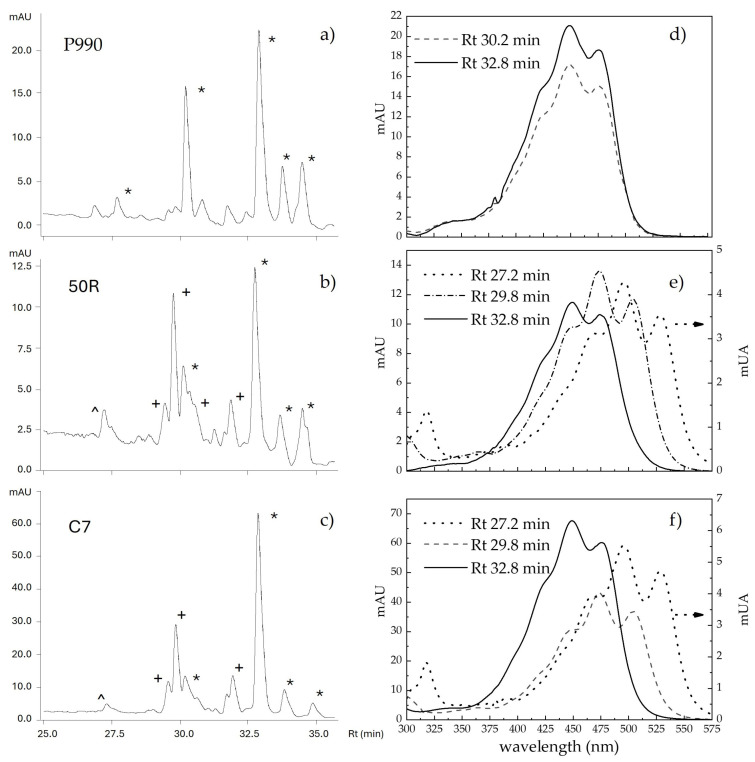
HPLC chromatograms of the methanolic extracts of *Cff* P990 (**a**), *Cff* 50R (**b**), and *Cff* C7 (**c**) pigments, after 14 days of growth on Cannellino naturalised medium. Different symbols mark the peak with different absorbance spectra corresponding to C.p. 450 (*), C.p. 473 (+), and C.p. 496 (∧) compounds. Spectra of the main peaks, acquired during elution in methanol, are reported in (**d**–**f**) for *Cff* P990, *Cff* 50R, and *Cff* C7, respectively.

The HPLC analysis of the bacterial methanolic extracts determined a series of compounds with different absorption properties, as reported in Table S1. Typical chromatograms of the pigments produced by *Cff* P990, *Cff* 50R, and *Cff* C7 strains when grown on Cannellino naturalised medium are shown in Figure 2a–c. Three main classes of carotenoids, C.p. 450, C.p. 473, and C.p. 496, formerly characterised in *Corynebacterium poisettiae* (reclassified as *Curtobacterium flaccumfaciens* (Hedges) [48]) cultures [42] were identified. According to the extinction coefficients reported by Norgård [42], we corrected the HPLC peak areas for the different absorbance response at 440 nm relative to that of the C.p. 450 compounds. The correction multiplicative factors were 1.77 and 1.24 for the C.p. 473 and C.p. 496 compounds, respectively. Accordingly, for the yellow *Cff* P990 strain, two main C.p. 450 peaks were found at retention times of about 30 min and 33 min. For the orange-red *Cff* 50R and *Cff* C7 strains, the main peaks corresponded to a C.p 473 compound at 29.8 min and to a C.p. 450 at around 33 min, as previously observed in *Curtobacterium flaccumfaciens* pv. *poinsettiae* [43]. The first, much smaller peak eluted at 27.2 min is considered a C.p. 496 compound, likely a bacterioruberin carotenoid.

In Figure 2d–f, the absorption spectra of the main eluted peaks, as well as that of the C.p. 496 compound, for *Cff* P990, *Cff* 50R, and *Cff* C7 strains grown on the Cannellino naturalised medium are reported. The three absorption bands observed for each class of compounds were centred at the following wavelengths regardless of the *Cff* strain: 424, 449, and 475 nm for C.p. 450; 449, 473, and 504 nm for C.p. 473; and 473, 497, and 526 for C.p. 496. For each *Cff* strain and culture medium, we can take into account the total relative contribution of carotenoids to the three classes of absorbers, C.p. 450, C.p. 473, and C.p. 496, as reported in Table 3. The *Cff* P990 strain produced only C.p. 450 compounds, on all the three growing media tested here, thus conferring the yellow colour to the *Cff* P990 colonies. In the case of *Cff* C7 and *Cff* 50R strains, their typical orange-red pigmentation is attributable to the combination between the large amounts of the C.p. 473 compounds with the minor, but significant, contribution of the C.p. 496, which shifted the total absorption to longer wavelengths.

### 3.2. Optical Characterisation of Naturalised Media

**Figure 3:**
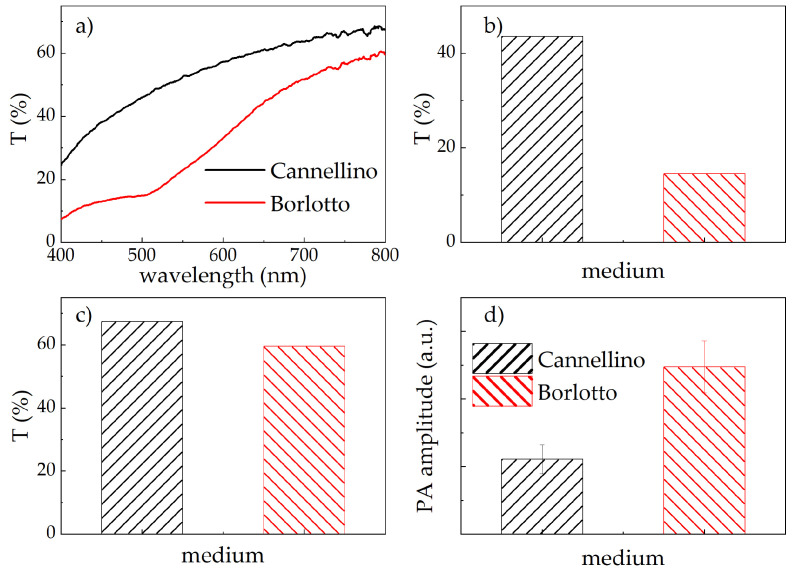
(**a**) Total transmittance spectra from the Cannellino- (black line) and the Borlotto-based media (red line). Comparison of the overall transmittance signal at 485 nm (**b**) and at 800 nm (**c**) between Cannellino- (black line) and Borlotto-based (red line) media. (**d**) Comparison of the PA amplitude between Cannellino- (black line) and Borlotto-based (red line) media excited by the whole laser white emission.

The previous analysis clearly showed that the pigmentation of each *Cff* strain leads to unique optical absorption properties. This suggests that these bacteria could be detected using optical techniques sensitive to absorption, such as Vis-NIR reflectance or photoacoustic spectroscopy. To verify this hypothesis, we developed the in vitro model mimicking a *Cff*-infected bean seed described in the Materials and Methods section. Firstly, the Cannellino and Borlotto naturalised media were tested as such, that is, uninoculated, to understand their potential influence on the overall optical response of the model system. The results are shown in Figure 3a–c. The Borlotto-based medium exhibited a drop in the total transmittance signal at around 510 nm, which indicates an absorption band overlapping the absorption region of the *Cff* strains (Figure 3a, red line). Additionally, the Borlotto-based medium showed a lower overall diffusive transmittance intensity (Figure 3b,c) in comparison with the Cannellino-based medium, indicating higher scattering and absorption properties. This can reduce the amount of excitation light reaching the *Cff* bacteria, thus weakening their potential detectable optical signals. Conversely, the Cannellino-based medium had a relatively flat total transmittance spectrum (Figure 3a, black line), without any specific spectroscopic feature.

### 3.3. Photoacoustic Characterisation

The optical characterisation of naturalised media confirmed that these materials are highly diffusive. Therefore, it appears difficult to detect the presence of bacterial pigments in vivo by Vis-NIR spectroscopy, as we can reasonably assume to have a similar diffusive condition observed in vitro in the whole bean seeds. At first, we analysed the *Cff* colonies embedded in the naturalised media to mimic their in vivo environment (Figure S1) by total reflectance measurements. The total reflectance spectra of our in vitro model (see Figure S5) showed only a minor spectral change at around 570 nm when comparing data from the uninoculated media (black lines) with those of media containing the *Cff* P990 colony (dashed lines). The Vis-NIR spectroscopy is less effective for detecting seed-borne bacteria, as it is unable to differentiate between light scattering caused by the host and light absorption caused by the bacteria.

Next, the same samples were characterised by using the PA setup. With a movable sample holder, we scanned the plates and measured the PA signals at different points. Initially, samples containing the uninoculated naturalised media were excited with the full laser emission range (e.g., 440–2400 nm). This excitation waveband was chosen to match as much as possible the large absorption bands of the target molecules in order to maximise the PA signal-to-noise ratio and also to detect PA contributions from other compounds present in the bacteria colonies and the media. According to the transmittance data (Figure 3a–c), the amplitude of the PA signal generated by the Borlotto-based medium was more intense when compared with that from the Cannellino-based medium (Figure 3d). Although, in principle, PA is proportional only to absorption, and it is insensitive to optical scattering, these data confirmed that the Borlotto-based medium has a significantly higher absorption than the Cannellino-based medium, across the entire laser emission range.

Then, PA signals from naturalised media embedded in the *Cff* colonies were measured. We found that the amplitude, period, and time of arrival to the transducer of the PA signal depended on the laser excitation point.

**Figure 4:**
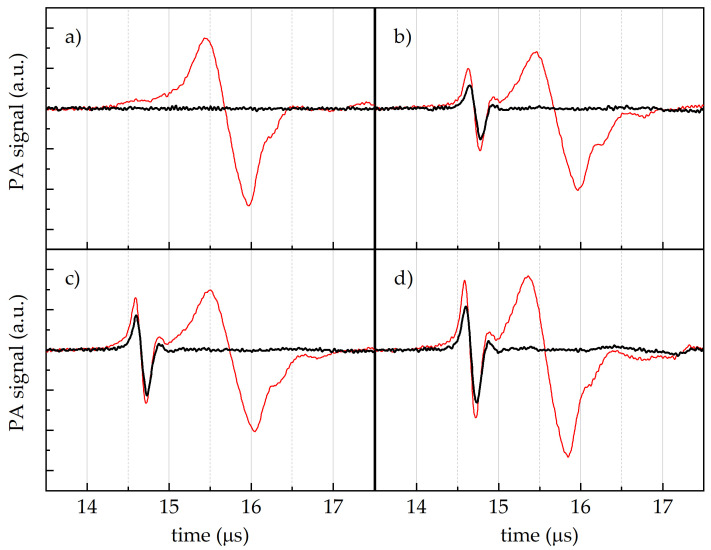
PA signal vs. time of arrival to the transducer from the Cannellino naturalised medium, after the excitation with the full laser emission (red lines), and with the laser emission filtered by the KG3 filter (black lines) at four different sample zones: (**a**) uninoculated point, (**b**) *Cff* P990 strain spot, (**c**) *Cff* 50R strain spot, and (**d**) *Cff* C7 strain spot.

In Figure 4, the PA amplitude signal vs. the time of arrival to the transducer from the Cannellino-based medium are reported as obtained after excitation with the full laser emission band (red lines) at an uninoculated zone (a), or in correspondence with the *Cff* P990 (b), *Cff* 50R (c), and *Cff* C7 (d) *Cff* strain spots. A single pulse at around 15.4 μs was recorded from the medium-uninoculated zone (Figure 4a). Two distinct pulses characterised the PA signals derived from the *Cff*-inoculated media, regardless of the *Cff* strain considered (Figure 4b–d, red lines): the first peak was at around 14.6 μs, while the second one was at around 15.4 μs.

When the near-infrared component of the laser was removed by means of the short-pass KG3 filter, the PA signal at longer times disappeared in all the samples, thus confirming that its origin was due to the contribution of water absorption in the near-infrared region (Figure 4, black lines). In contrast, the shorter-time pulse was absent in the medium spot (Figure 4a), while it was still present in the spots of the *Cff* colonies (Figure 4b–d, black lines), thus confirming its specific attribution to the absorbance of *Cff* bacteria.

**Figure 5:**
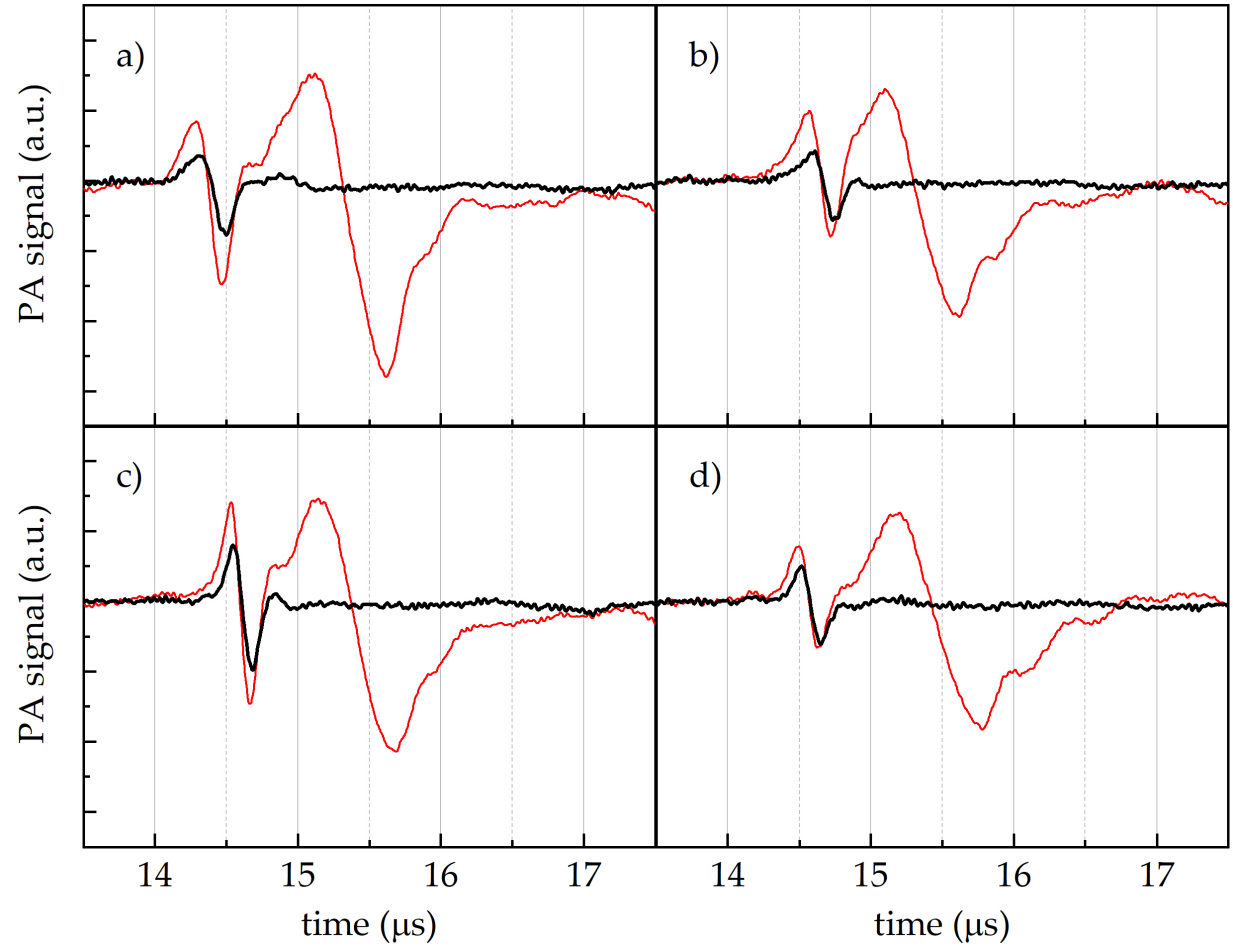
PA signal vs. time of arrival to the transducer from the Borlotto naturalised medium, after the excitation with the full laser emission (red lines), and with the laser emission filtered by the KG3 filter (black lines) at four different sample zones: (**a**) uninoculated point, (**b**) *Cff* P990 strain spot, (**c**) *Cff* 50R strain spot, and (**d**) *Cff* C7 strain spot.

The same experiments were replicated on the Borlotto-based medium. The results obtained after excitation with the full laser emission band are shown in Figure 5 as red lines. In this case, the PA signal was composed of two pulses in each point of the sample. After filtering the near-infrared component of the laser, the longer-time PA signal disappeared in all the samples, thus again confirming that its origin was due to the contribution from water absorption. In contrast, the shorter-time pulse was still present in all the zones (Figure 5, black lines), so it was not exclusively related to the presence of *Cff*, as it was with the Cannellino-based medium. These data could be partly expected, since it was shown that the Borlotto-based medium absorbs light in a spectral region similar to that of the *Cff* pigment absorbance (Figure 3), thus making it more difficult to detect and distinguish the *Cff* signal alone.

**Figure 6:**
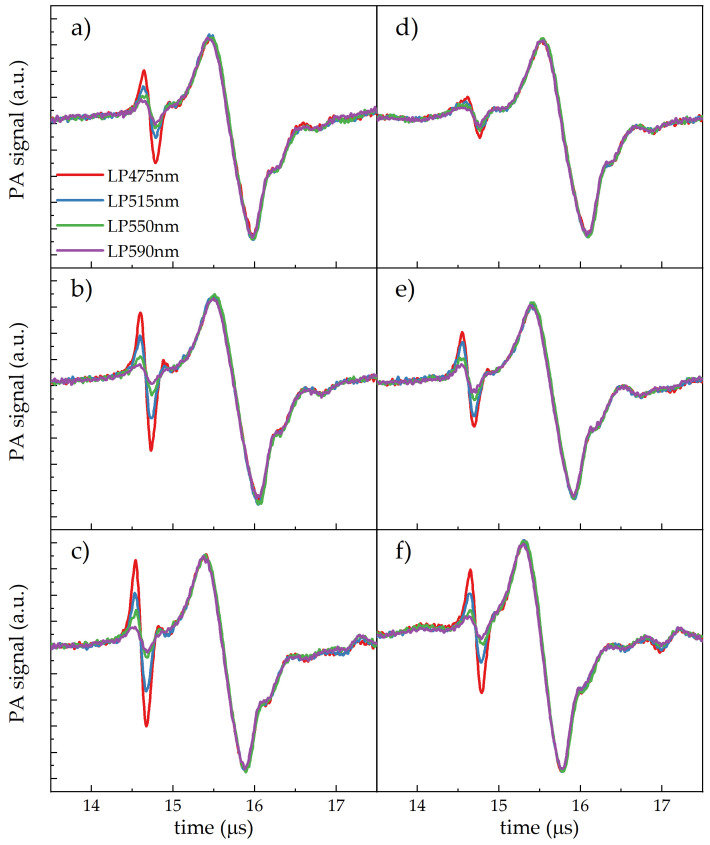
PA signal vs. time of arrival at different excitation wavebands defined by the laser emission spectrum combined with long-pass (LP) filters (at cut-on wavelengths of 475, 515, 550, and 590 nm) from spots of the P990 (**a**,**d**), 50R (**b**,**e**), and C7 (**c**,**f**) *Cff* strain grown in the Cannellino (**a**–**c**) or Borlotto naturalised media.

To further characterise the PA signals recorded on the *Cff* colonies, the laser excitation waveband was modified by means of long-pass filters with cut-on wavelengths from 475 nm to 630 nm. The acquired signals for the three *Cff* strains in both Cannellino- and Borlotto-based media are shown in Figure 6.

For all the *Cff* strains in both media, the PA amplitude of the signal at 14.6 μs decreased as the waveband of the excitation spectrum was moved from the blue to the red, while the water-related PA signal was unaffected. PA measurements after filtering the near-infrared water contribution are not reported, since the 20% attenuation introduced by the KG3 short-pass filter in the visible range excessively reduced the bacterial signal under the higher excitation wavebands (LP550, LP590).

## 4. Discussion

Here, we demonstrated that the *Cff* bacterial pigmentation depends on both the strain and the growth medium (Table 2). The analysis of the pigment composition (Figure 2, Table S1 of Supplementary Materials) excludes the presence of both β-carotene and lycopene C40 carotenoids (retention time of 34.9 min and 37.6 min for lycopene and β-carotene, respectively). Different peaks with the same absorption spectrum and different polarity may represent mono- and di-glycoside compounds of the same chromophore, as glycosylation does not change its absorbance spectral shape [49].

The *Cff* pigment composition was rich in C50 carotenoids, as identified early [42]. They possess higher antioxidant properties than C40 carotenoids, such as β-carotene [50]. C50 carotenoids are synthesised only in few bacterial species and appear abundant in the coryneform bacterial groups [51], in which they occur in a constitutive, light-independent manner [52].

The main reason inducing the synthesis of carotenoids in non-photosynthetic bacteria, such as *Cff*, is to protect themselves by stress-generated reactive oxygen species (ROS). In the C50 carotenoids, light absorption at longer wavelengths (lower energy of the excited state) can favour the physical quenching of singlet oxygen. Furthermore, the presence of hydroxyl groups at the ends of carotenoids can strengthen their binding to biological membranes [53], providing them with membrane stability and bacterial cell resistance to abiotic stresses [54]. In addition, carotenoids represent a good substrate for oxidants and free radicals, leading them to the protection of the organisms. However, the relationship between the synthesis of a specific class of carotenoids and a particular growth medium remains unclear.

Our findings indicate that the synthesis of carotenoid compounds gives each *Cff* strain distinct optical absorption properties in the blue-green region, with specific peaks that vary in shape and intensity based on the carotenoid composition (Figure S2). These features were exploited as a preliminary step in the development of a non-destructive PA technique able to detect seed-borne pathogens in infected bean seeds. The capability of our PA technique to catch the presence of *Cff* in a mimicked-bean seed was clearly demonstrated (Figure 4). By using a white light source, we could show the detection of two temporally separate PA signals, with the earlier one coming from the bacterial pigment absorbance around 500 nm and the latter from the IR absorption peaks of water (the primary background signal, Figure S4a).

The time of arrival to the transducer of the PA signals, considering the speed of sound in water (i.e., 1482 m $s^{-1}$ [45]), allowed the calculation of the distance between the absorber, which generated the PA signal, and the transducer. In our case, the first pulse was generated at around 2.1 cm from the US receiver, while the second one went from around 2.3 cm, i.e., the first pulse originated from a point closer to the transducer than the second pulse. Indeed, according to our setup (Figure S3a), the laser beam initially interacted with the medium+water layer and successively irradiated the bacterial spot layer. The distance difference estimated from the two arrival times (approximately 2 mm) was consistent with the thickness of the *Cff* colony layer. Moreover, the shorter period of the first pulse with respect to the second one suggested a more localised optical absorber.

**Figure 7:**
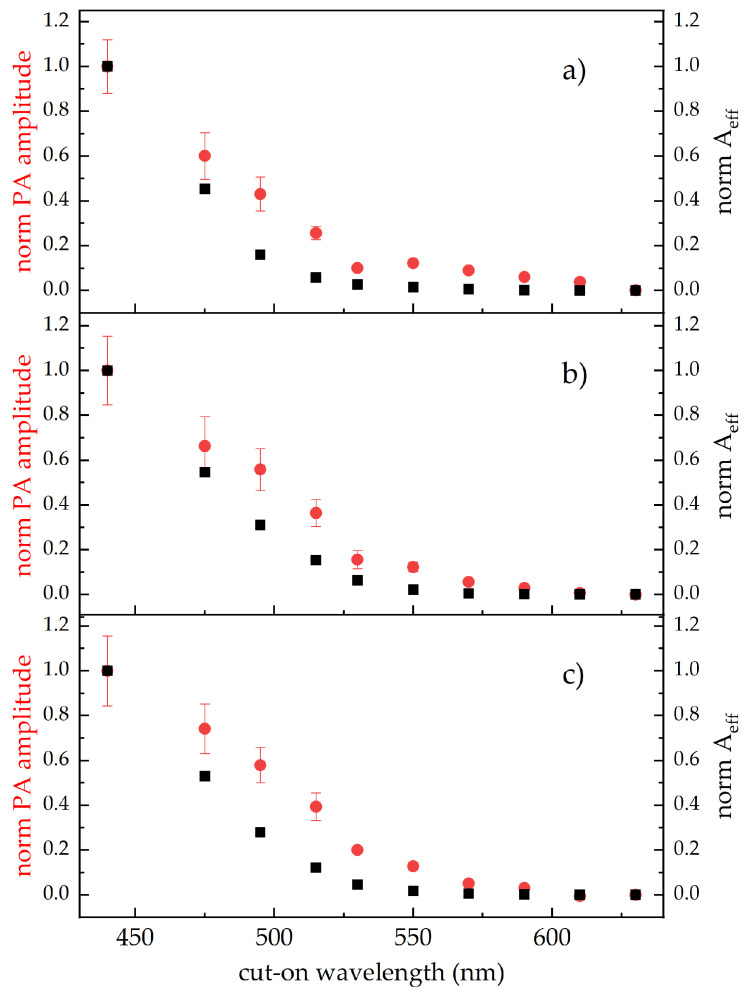
(**a**–**c**) Amplitude of the bacterial PA signal (red circles) and the $A_{\mathrm{eff}}$ (black squares) of *Cff* pigments as a function of the excitation waveband. Wavebands are identified by the cut-on wavelengths of the long-pass filters from 475 nm to 630 nm. Each set of data is normalised to the first point at 440 nm corresponding to the measurement with the whole laser emission excitation.

The kind of dependence of the PA signals on the excitation waveband (Figure 6) provided further support to the assignment of their origin. Moving the irradiation from blue to red wavelengths, there is a reduced overlapping of excitation on the absorbance of the *Cff* carotenoid compounds, leading to a proportional decrease in the PA signal amplitude. This effect is more clearly evidenced in Figure 7, in which the PA amplitude as a function of the excitation waveband for the three *Cff* strains in the Cannellino medium is compared to the effective absorbance ($A_{\mathrm{eff}}$) of the bacterial carotenoids.

For each strain, the PA amplitude and the $A_{\mathrm{eff}}$ showed a similar trend as a function of the excitation waveband, even if the $A_{\mathrm{eff}}$ decrease was steeper than that of the PA amplitude. This divergence is likely due to the utilisation of the available in vitro pigment absorption spectra in calculating $A_{\mathrm{eff}}$ (Equation (1)), considering that a significant difference between the in vivo and the in vitro carotenoid absorption spectra occurs [55]. The in vivo interaction of carotenoids with biological membranes can favour pigment aggregation that induces a large increase in absorption in the yellow-red spectral region with respect to that of carotenoids in solvent solutions [56,57].

Therefore, if available, using the in vivo carotenoid absorption spectrum to calculate the $A_{\mathrm{eff}}$ of bacterial carotenoids would produce a better agreement between the PA amplitude and the target molecule absorbance as changing the excitation wavelength, with respect to that shown in Figure 7.

The dependence of the PA signals on the excitation waveband in accordance with the absorbance of the *Cff* pigments further confirms the capacity of our PA technique to detect the presence of bacteria spots inside the mimicked infected-bean samples.

Furthermore, our data indicate that by selecting specific excitation wavelengths, it is possible to discriminate different *Cff* strains. In particular, we can define a PA excitation ratio between the amplitude recorded with the LP515 (PA_515_) and that recorded with the full laser band (PA_440_), which showed values for the orange-red *Cff* C7 and 50R strains 1.5 times higher than that of the *Cff* P990 strain. These kinds of indices could be of particular interest since it was reported than orange strains are much more virulent then yellow ones [37].

The above evidence was collected on samples of *Cff* grown in a highly diffusive but low-absorbing medium like the Cannellino medium. Conversely, our results showed that distinguishing the pigment PA signal (i.e., bacteria) from that of an absorbing medium like Borlotto medium (Figure 5, black lines) is more challenging.

To address this limitation, more investigation is needed by testing specific laser excitation wavelengths as the best compromise between penetration within the medium, the bacterial pigment absorption, and the intensity of the acoustic signal generated in order to discriminate distinct components from the growth medium.

Furthermore, using a PA setup like ours, based on the direct detection of the ultrasonic waves induced by the laser [33,34,35], gives the advantage to detect the position of the absorbers, by the analysis of the arrival time of the PA signals to the transducer, which is crucial for distinguishing between different PA contributions.

## 5. Conclusions

In conclusion, our optical characterisation of *Cff* bacteria revealed the complexity of their pigmentation that demands further investigation to better understand its origin and relationship with strains and growing media.

Nonetheless, we have successfully shown, for the first time, that *Cff* bacteria embedded in a mimicked infected-bean model produces a PA signal detectable through the measurement of laser-induced ultrasound waves using a direct PA configuration [33]. Unlike traditional PA spectroscopy, this experimental setup offers greater flexibility in the sample analysis, as it is non-contact and allows real-time monitoring. It also enables the discrimination between two classes of *Cff* strains, the yellow- and the orange-red-coloured strains, which possess different virulence.

This initial step demonstrated the usefulness of the proposed PA setup as a technique for monitoring seed-borne bacteria in in vitro studies. It could allow time-course measurements of the bacterial colony development under different growing media. X-Y-scanning the samples could quantify the size and homogeneity of the bacterial spots producing 2D images of it and also quantify the volume of the colony, determining the colony thickness.

Although our study was in a preliminary stage, it revealed the potential of this configuration for the in vivo detection of seed-borne bacteria. In perspective, a quantitative estimation of the bacterial burden could be achieved by calibrating the intensity of the PA signal at specific excitation wavelengths to determine the concentration of different bacterial strains.

These findings could help in defining methods for preventing or limiting the *Cff* proliferation, which remains a global threat to worldwide legume production, contributing to improve crop health and food security.

## Figures and Tables

**Table 1:** *Cff* strain used in this study and their main features.

Strain	Synonym	Pigmentation	Year and Country	Host
*Cff* P990	CFBP8820 ICMP22053	Yellow-fluidal	2015, Iran	*Capsicum annuum*
*Cff* 50R	CFBP8819 ICMP22071	Red-fluidal	2014, Iran	*Phaseolus vulgaris*
*Cff* C7	J24	Orange-fluidal	2004, USA	*Phaseolus vulgaris*

**Table 2:** Pigmentation of *Cff* strains used in this study on different growth media.

Strain	LB	LBsac10	Cannellino	Borlotto
P990	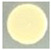	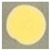	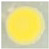	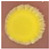
50R	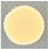	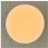	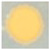	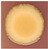
C7	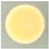	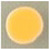	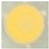	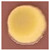

**Table 3:** Total relative (%) contribution of different classes of carotenoids (Car. Class) produced by the tested *Cff* strains on LB, LBsac10, and Cannellino naturalised medium (Cann).

Car. Class	P990	P990	P990	50R	50R	50R	C7	C7	C7
	LB	LBsac10	Cann	LB	LBsac10	Cann	LB	LBsac10	Cann
C.p. 450	80.1	90.3	92.6	38.2	25.3	36.0	45.6	35.5	47.0
C.p. 473				36.9	59.9	53.3	47.7	57.0	42.2
C.p. 496				4	4.8	2.7		1.7	1.8

## Data Availability

The raw data supporting the conclusions of this article will be made available by the authors on request.

## Supplementary Materials

The following are available online at https://www.mdpi.com/article/10.3390/s24237616/s1. Figure S1: Cannellino- and Borlotto-based medium embedding *Cff* strains for PA experiments. Figure S2: Absorption spectra of the *Cff* pigment methanolic extracts for the P990 (black line), 50R (blue line), and C7 (red line) strains, normalised to their maximum. Figure S3: (a) Sketch of PA setup. (b) Typical PA signal in the time domain. Figure S4: (a) Left y-axis: absorption spectra of *Cff* bacterial colonies (P990, black line; 50R, blue line; C7, red line) and of water (green line [58]). Right y-axis: transmittance of KG3 filter (orange line) and emission band from laser (black dotted line). (b) Left y-axis: transmittance spectra of long-pass filters for several cut-on wavelengths (solid lines) and of KG3 filter (dashed line). Right y-axis: emission band from laser (black dotted line). Figure S5: Total reflectance spectra of uninoculated (a) Cannellino- and (b) Borlotto-based medium (black lines) or embedding a *Cff* P990 colony (dashed lines). Table S1: Relative (%) content of the main carotenoid compounds produced by the different strains of *Cff* in various culture media determined by the HPLC analysis of bacterial extracts. Reference [58] is cited in the supplementary materials.
