# Supplementary material for: Exploiting Bacterial Pigmentation for Non-Destructive Detection of Seed-Borne Pathogens by Using Photoacoustic Techniques
Source: Sensors (Basel). 2024 Nov 28;24(23):7616. doi: 10.3390/s24237616 (PMC11644776; doi:10.3390/s24237616)
Supplement: Supplementary file 1 [file sensors-24-07616-s001.zip › sensors-3313598-supplementary.pdf]

Supplementary Materials

Exploiting Bacterial Pigmentation for non-Destructive Detection of Seed-borne Pathogens by Using Photoacoustic Techniques

Lucia Cavigli[1,‡], Dario Gaudioso[2,‡], Cecilia Faraloni[3], Giovanni Agati[1,*] and Stefania Tegli[2,*]

1 Consiglio Nazionale delle Ricerche, Istituto di Fisica Applicata "Nello Carrara", Via Madonna del Piano 10, 50019 Sesto Fiorentino, Italy
2 Dipartimento di Scienze e Tecnologie Agrarie, Alimentari, Ambientali e Forestali (DAGRI), Università degli Studi di Firenze, Piazzale delle Cascine 18, 50144 Firenze (FI), Italy
3 Consiglio Nazionale delle Ricerche, Istituto per la BioEconomia, Via Madonna del Piano 10, 50019 Sesto Fiorentino, Italy
* Correspondance: g.agati@ifac.cnr.it; stefania.tegli@unifi.it;
‡ These authors contributed equally to this work.

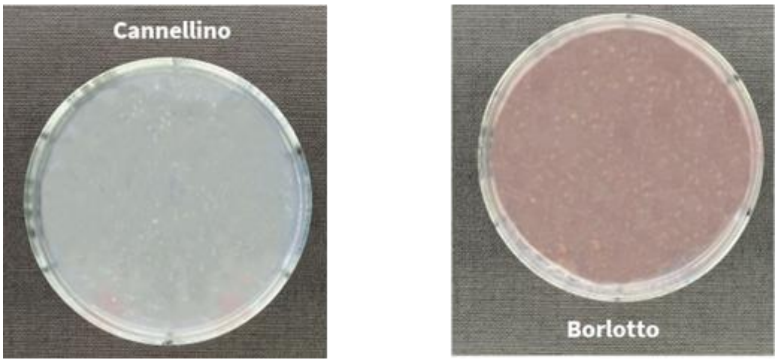

Figure S1 Cannellino- and Borlotto-based medium embedding *Cff* strains for PA experiments.

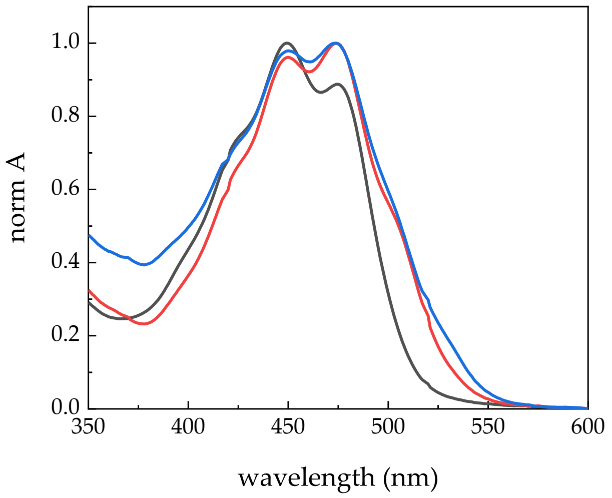

Figure S2 Absorption spectra of the *Cff* pigment methanolic extracts for the P990 (black line), 50R (blue line), and C7 (red line) strains, normalized to their maximum.

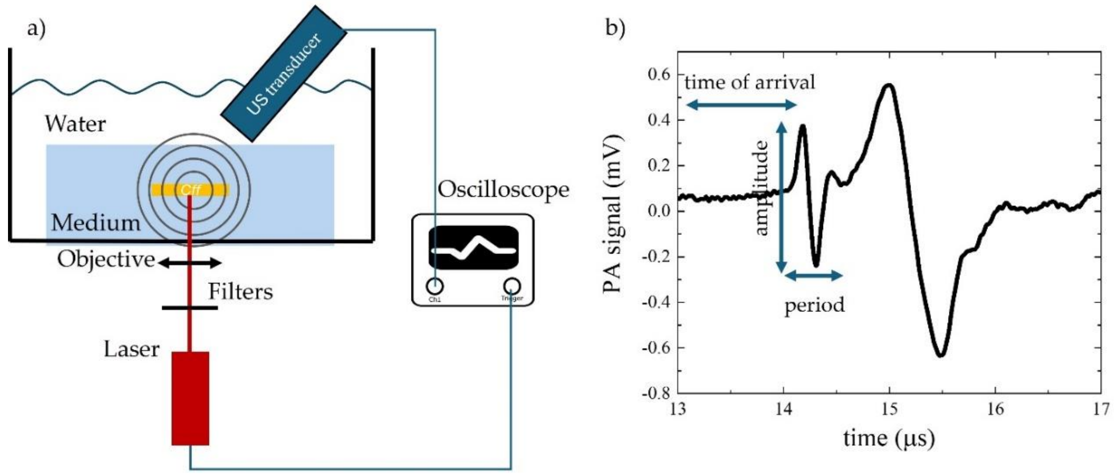

Figure S3 a) Sketch of the PA-Setup. b) Typical PA signal in the time domain.

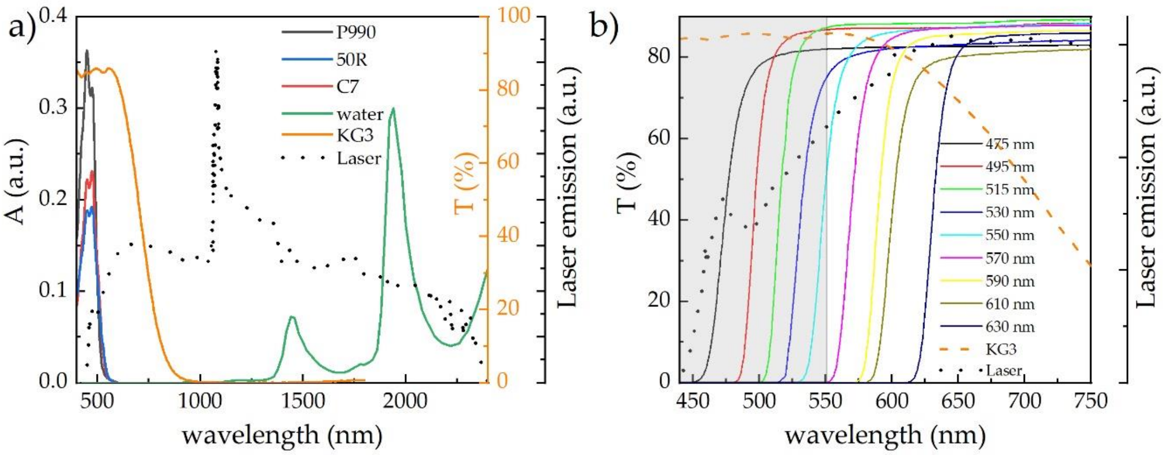

Figure S4 a) Left y axis: absorption spectra of *Cff* bacterial colonies (P990 black line, 50R blue line, C7 red line), and of water (green line [1]). Right y axes: transmittance of KG3 filter (orange line) and emission band from laser (black dotted line). b) Left y axis: transmittance spectra of longpass filters for several cut-on wavelengths (solid lines), and of KG3 filter (dashed line). Right y axis: emission band from laser (black dotted line). The grey area indicates the spectral range of *Cff* absorbance.

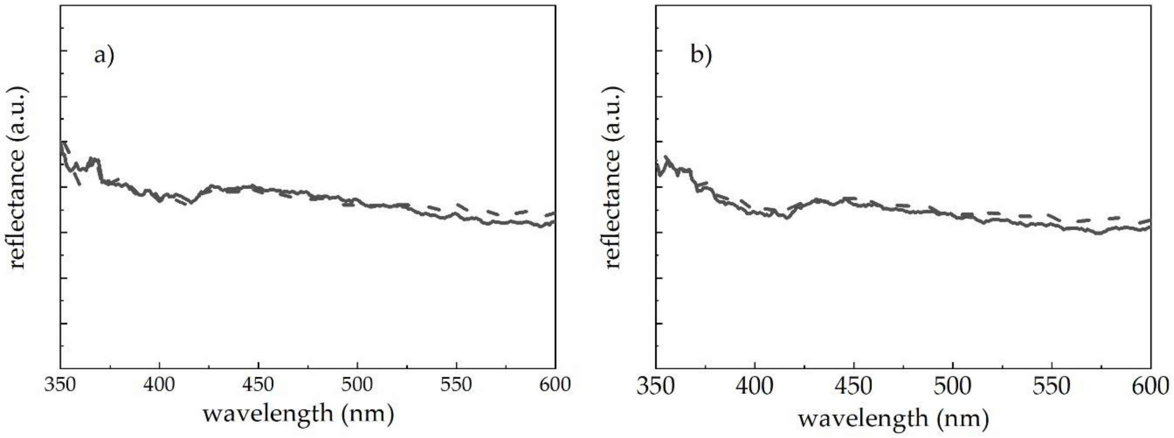

Figure S5. Total reflectance spectra of a) Cannellino- and b) Borlotto-based uninoculated medium (black lines) or embedding a *Cff* P990 colony (dashed lines).

References

1. Hale, G. M.; Querry, M. R. Optical constants of water in the 200 nm to 200 μm wavelength region. *Appl. Opt*. **1973**, *12* 555-563.

Table S1. Relative (%) content of the main carotenoid compounds produced by the different strains of *Cff* in various culture media determined by the HPLC analysis of bacterial extracts*,†.

Cff			**P990**	**P990**	**P990**		**50R**	**50R**	**50R**		**C7**	**C7**	**C7**
			LB	LBsac10	Cann		LB	LBsac10	Cann		LB	LBsac10	Cann
Carotenoid Class	Rt (min)												
C. p. 496	27.2						4.0	4.8	2.7			1.7	1.8
C. p. 473	27.5							42.7				8.3	
C. p. 450	27.7			30.4	3.8			6.5					
C. p. 473	29.6						28.6		6.7		23.4	13.2	8.7
C. p. 473	29.8							17.2	19.8			25.8	23.1
C. p. 450	30.1		47.9						6.7				
C. p. 450	30.2			57.3	18.8			16.7				22.3	7.6
C. p. 473	30.3						8.3		14.6		9.2		
C. p. 450	30.7		13.8		5.3								
C. p. 450	31.7				3.6								
C. p. 473	31.9								12.2		15.1	9.7	10.4
C. p. 450	32.6				2.9		9.0						
C. p. 450	32.8		18.4	2.6	34.2		7.0	2.1	18.0		28.6	13.2	34.5
C. p. 450	33.5												
C. p. 450	33.7				10.3				5.3		6.0		4.9
C. p. 450	34.5				13.7		4.3		6.0		11.0		
C. p. 450	35.9						17.9						

*Contributions of the C.p. 473 and C.p. 496 compounds were corrected for their absorbance response at 440 nm relative to that of C.p. 450.

†Elution time for the lycopene and β-carotene standards was 34.9 min and 37.6 min, respectively.
